# Spin in dental publications: a scoping review

**DOI:** 10.1590/1807-3107bor-2024.vol38.0065

**Published:** 2024-07-15

**Authors:** Laís Rueda CRUZ, Stephanie Fumagalli BRAGA, Paulo NADANOVSKY, Ana Paula Pires dos SANTOS

**Affiliations:** (a) Universidade do Estado do Rio de Janeiro – UERJ, School of Dentistry, Department of Community and Preventive Dentistry, Rio de Janeiro, RJ, Brazil.; (b) Universidade do Estado do Rio de Janeiro – UERJ, Institute of Social Medicine, Department of Epidemiology, Rio de Janeiro, RJ, Brazil.

**Keywords:** Evidence-Based Dentistry, Oral Health, Review, Bias, Research Report

## Abstract

The aim of this review was to map the practice of spin in scientific publications in the dental field. After registering the review protocol (osf.io/kw5qv/), a search was conducted in MEDLINE via PubMed, CENTRAL, Embase, Scopus, LILACS, ClinicalTrials.gov, and OpenGrey databases in June 2023. Any study that evaluated the presence of spin in dentistry was eligible. Data were independently extracted in duplicate by two reviewers. After removing duplicates, 4888 records were screened and 38 were selected for full-text review. Thirteen studies met the eligibility criteria, all of which detected the presence of spin in the primary studies, with the prevalence of spin ranging from 30% to 86%. The most common types of spin assessed in systematic reviews were failure to mention adverse effects of interventions and to report the number of studies/patients contributing to the meta-analysis of main outcomes. In randomized controlled trials, there was a focus on statistically significant within-group and between-group comparisons for primary or secondary outcomes (in abstract results) and claiming equivalence/noninferiority/similarity for statistically nonsignificant results (in abstract conclusions). The practice of spin is widespread in dental scientific literature among different specialties, journals, and countries. Its impact, however, remains poorly investigated.

## Introduction

Spin or distortion bias is the intentional or unintentional use of any strategy that distorts the reporting of the results of a study to overestimate the beneficial effect or ignore and minimize side/adverse effects, uncertainties, and disadvantages of an intervention.^1-7^ It can be motivated by a variety of reasons, such as the urge to impact science, author’s ignorance, financial gains, unconscious bias, or simply the desire to mislead the reader. However, regardless of the motivation, spin favors the author’s interest, be it intellectual, financial, or academic.^1-6^

Spin can be present in different forms. The distortion of methods includes modifications to the protocol, such as changes in the objective and hypothesis or the “beautification” of the description of the methods. Results may be distorted by reporting partial or incomplete data, by emphasizing statistically significant results even when they are not related to the primary outcome, or by focusing on another objective that yielded a statistically significant result to shift the reader’s attention from nonsignificant results. Spin can also be detected as an interpretation of data that is incompatible with the results of the study.^3-5^ Finally, the rhetoric or the way an article is written, using biased and persuasive language, may lead the reader to misinterpret the findings.^3,4^

Spin can be found in different study designs^1,2,8,9^ and in any section of an article, from title and abstract to full text.^1^ In the case of randomized controlled trials (RCTs) with nonsignificant results, an analysis of 72 articles found that more than 40% of them had spin in the full text (results, discussion, or conclusion) and approximately 60% showed some type of spin in the abstract conclusion.^1^

The consequences of using spin are concerning. The main concern is the misinterpretation of results by readers, both professionals and patients.^4^ Oncologists who read abstracts with spin were more likely to believe in the benefit of a treatment than those who read the rewritten abstracts without spin,^10^ which could put several patients’ lives at risk. Another consequence of spin is associated with media coverage and news reports.^11,12^ Patients and the general public, especially those searching for new therapies and drugs, are more likely to believe the results of studies reported with spin.^13^ Therefore, spin could result in manipulation and misinformed choices.^7^ In addition, misinterpretation of the results of a study can raise suspicions about new treatments and influence policymakers to approve unsuitable regulations and policies.^7,12^

Thus, it is opportune to identify, characterize, and quantify spin within the dental literature. As such, this scoping review aimed to map the practice of spin in scientific publications in dentistry.

## Methods

We followed the Joanna Briggs Institute (JBI) methods for scoping reviews^14^ and reported the review according to PRISMA Extension for Scoping Reviews (PRISMA-ScR).^15^

### Protocol and registration

The protocol of this review was prospectively registered on the Open Science Framework on 22 February 2022 (available at https://osf.io/kw5qv/).

### Eligibility criteria

The eligibility criteria were developed using a PCC framework (Participants, Concept, Context).^14^ Participants were any study that reported spin in the dental literature with no language, date, or study design restrictions. Concept was the presence of spin in any section of the publication (e.g., abstract, results, and discussion), and context was any dental publication defined as follows: article published in dental journals or dental grey literature; or article not published in dental journals or dental grey literature that is within the field of dentistry and has at least a dentist or a dental researcher affiliated with a dental school listed as one of the authors.

### Information sources

The search strategy was designed to identify both published and unpublished studies. A preliminary search was undertaken in MEDLINE to identify articles on the topic. The text words contained in the titles and abstracts of relevant articles and the index terms used to describe these articles were used to develop a sensitive search strategy for MEDLINE via PubMed, CENTRAL, Embase, Scopus, and LILACS. The reference lists of all included sources of evidence were screened for additional studies. Sources of grey literature, unpublished, and ongoing studies included OpenGrey (www.opengrey.org) and ClinicalTrials.gov (www.clinicaltrials.gov). The search was conducted in June 2023 with no language, date, or study design restrictions.

### Search strategy

The final search conducted in MEDLINE via PubMed is available in Supplemental file I (osf.io/kw5qv/). The search strategy, including all identified keywords and index terms, was adapted for each included database and/or information source. The terms selected to represent spin were: “spin”, “distort*”, “misinterpret*”, “misreport*”, “mislead*”, “misrepresent*”, and “extrapol*”. To cover all areas of dentistry, the terms “dent*”, “oral health”, and all Medical Subject Headings (MeSH) terms referring to dental specialties were included, such as endodontics and periodontics.

### Selection of sources of evidence

All records found were added to EndNote® (Clarivate Analytics, Philadelphia, USA). After removal of duplicates, the records were exported to Rayyan software,^16^ where titles and abstracts were independently screened by two reviewers. Potentially eligible records were read in full and reasons for exclusion were recorded. Disagreements were resolved by a third reviewer.

### Data charting process

Data from the included studies were independently extracted by two reviewers using a data extraction form developed for this study and previously piloted, available as Supplemental File II (osf.io/kw5qv/). Any disagreement was solved by consensus among the reviewers.

### Data items

The extracted data included details about the participants, concept, context, authors’ country, journal, publication date, dental area, section where spin was assessed (title and abstract, full text, or both), study design, results, conclusions, and funding.

### Synthesis of results

The search results were presented in a flow diagram (Figure).^15^ After data extraction, the results of all included studies were narratively described.

**Figure:**
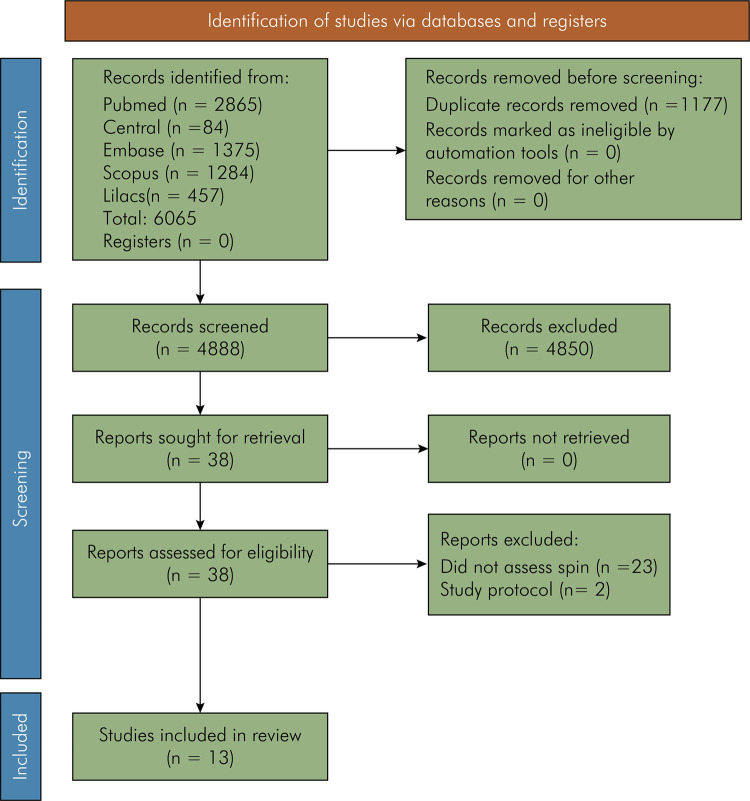
Flow diagram showing the selection of sources of evidence.

## Results

### Selection of sources of evidence

The electronic search yielded a total of 6065 records. No studies were identified from hand searching or grey literature. After removing 1177 duplicates in EndNote® (Clarivate Analytics, Philadelphia, USA), 4888 records were exported to Rayyan where titles and abstracts were scanned. Of these, 38 were considered potentially eligible and selected for full-text review. Twenty-five studies were excluded for the following reasons: 23 did not assess spin and two were protocols. Thirteen studies met the inclusion criteria and were included.^17-29^(Figure).

### Characteristics of sources of evidence

The studies were published from 2017 to 2023 and covered the following dental specialties: implantology,^17,26,29^ endodontics,^19,28,29^ orthodontics,^21,22^ periodontics,^24,26^ operative dentistry,^20,25^ cariology,^23^ oral surgery,^29^ oral oncology,^29^ general dentistry,^18,29^ and dentofacial trauma.^27^ The 13 studies were from the following countries: Brazil,^17^ Switzerland,^18,22^ China,^19-21,26,27^ Greece,^28^ Germany,^23^ United States of America,^24,29^ and the Netherlands.^25^

### Results of individual sources of evidence

Ten studies assessed the presence of spin in RCTs,^18-24,26,27,29^ whereas three studies assessed spin in systematic reviews (SRs).^22,25,28^ Only one overview^17^ was identified evaluating the presence of spin in SRs. In most studies spin was only assessed in abstracts,^19-21,23,24,26,27,29^ while in four studies spin was also assessed in the full text.^17,18,25,28^ All studies detected spin in the evaluated studies, with prevalence ranging from 30%^7^ to 86%.^9^

In those studies investigating the presence of spin in SRs,^17,22,25,28^ two types of spin were most common: failure to report adverse effects of interventions^17,25,28^ and failure to report the number of studies/patients actually contributing to the meta-analysis of main outcomes.^22,28^ In the studies that evaluated the presence of spin in RCTs, the most common spin in the abstract results was a focus on statistically significant within-group and between-group comparisons for primary or secondary outcomes,^19-21,24,26^ and in the abstract conclusion was claiming equivalence/noninferiority/similarity for statistically nonsignificant results.^19-21^

### Synthesis of results

Data from the included studies are summarized in the Table.

**Table:** Characteristics of the included studies.

Author/Year	Country	Journal	Dental area	Study section where spin was assessed	Study design where spin was assessed	Most frequent spin/prevalence (%)
Lucena, 2022	Brazil	Clinical Implant Dentistry and Related Research	Oral implantology	Abstract and full text	SR	Failure to mention adverse events of interventions in the abstract (51%) and full text (27%).
Eleftheriadi, 2020	Switzerland	Journal of Dentistry	General dentistry	Abstract and full text	RCT	Abstract: types 1 and 2 and types 1 and 3 (38% each combination).*
						Full text: types 1 and 4 (24%).*
Fang, 2020	China	International Endodontic Journal	Endodontics	Abstract	RCT	Results: Emphasizing statistically significant results within groups (12%).
						Conclusion: Claiming equivalence for statistically nonsignificant primary outcomes (27%).
Fang, 2022	China	Operative Dentistry	Operative dentistry	Abstract	RCT	Results: Focusing on significant within-group comparisons for primary outcomes (22%).
						Conclusion: Claiming equivalence/noninferiority/comparability/similarity for statistically nonsignificant results (21%).
Fang, 2023	China	Dental Traumatology	Dentofacial trauma	Abstract	RCT	Results: Focus only on time points with statistical significance when multiple time points for primary outcomes existed (13.3%).
						Conclusion: Focus on statistically significant results (i.e., secondary outcomes, subgroup analysis, and within-group analysis) (23.3%).
Giannakoulas, 2022	Greece	International Endodontic Journal	Endodontics	Abstract and full text	SR	Failure to report the number of patients/teeth/studies contributing to meta-analysis was identified in 80 of 125 abstracts with spin (64%).
Guo, 2021	China	European Journal of Orthodontics	Orthodontics	Abstract	RCT	Results: Focusing on significant within-group comparisons for primary outcomes (42%).
						Conclusions: Claiming equivalence or noninferiority for statistically nonsignificant results (51%).
Makou, 2021	Switzerland	European Journal of Orthodontics	Orthodontics	Abstract	SR	Failure to report the number of studies and patients contributing to the meta-analysis of the main outcome (68%).
Reda, 2017	Germany	Community Dentistry and Oral Epidemiology	Cariology	Abstract	RCT	30% (13) of the papers were found to have spin, that is, unsubstantiated claims. Does not mention the type of spin most found.
Roszhart, 2019	United States of America	The Journal of the American Dental Association	General dentistry, dental research, oral implantology, endodontics, oral surgery, periodontology, oral oncology	Abstract	RCT	Concluding clinical significance despite no statistical significance (23%) and interpreting statistically nonsignificant results for the primary outcome as showing treatment equivalence or comparable effectiveness (23%).
Sensever, 2022	Netherlands	Journal of Dentistry	Restorative dentistry	Abstract and full text	SR	Abstract: Reporting of adverse effects of the interventions (65%).
						Full text: Recommendations for clinical dental practice not supported by the findings (22%).
Shaqman, 2020	United States of America	PLOS ONE	Periodontics	Abstract	RCT	No definition of primary or secondary outcomes (79%).
Wu, 2020	China	Journal of Clinical Periodontology	Periodontology, oral implantology	Abstract	RCT	Results: Focusing on secondary outcomes (16%).
						Conclusion: Focusing on within-group comparisons (29%).

RCT: randomized controlled trial; SR: systematic review. * 1 – Focus on statistically significant results; 2 – Claim equivalence or comparable effectiveness for nonsignificant results; 3 – Claim beneficial effect of nonsignificant results; 4 – Other (when a p-value fails to reach a significant threshold, authors may imply a “trend toward statistical significance” or otherwise suggest that the failure to achieve statistical significance is due to insufficient data).

## Discussion

We identified 13 studies investigating spin in dental publications from 2017 to 2023. Each of these 13 secondary studies detected spin in their primary studies, covering different dental areas. The most conservative (lowest) estimate of prevalence of spin in the dental literature was 30%, whereas one study found a prevalence of 86%.

Although the term spin was firstly described in 1995,^30^ studies evaluating its occurrence in the scientific literature are quite recent. The first study we identified on this topic was published in 2010 and described forms of spin in medical articles, while also being the first study to suggest a classification of the type and extent of spin for titles, abstracts, and main texts of RCTs.^1^ In dentistry, the first study assessing the presence of spin was published in 2017. In this study, spin was a secondary analysis with no attempt to identify its type or severity.^23^

Most of the studies included in our scoping review only assessed the presence of spin in the abstracts.^19-24,26,27,29^ The main reason for assessing only the abstracts is that it is the only section of an article most readers choose to read due to lack of time, information overload, or difficulty accessing the article’s full text.^31^ Regardless of the growth in the open access movement,^32^ it is estimated that only 28% of published articles are freely available online.^33^ Also, abstracts provide a “first impression,” allowing readers to gauge their interest in the study, so it is essential that abstracts are well written. Abstracts of RCTs are especially important because many health professionals base their treatment choices on them.^34^ In an attempt to standardize abstracts, the Consolidated Standards of Reporting Trials (CONSORT) has promoted a specific guideline for writing RCT abstracts since 2008.^31^ However, improvements in abstract writing remain unsatisfactory.^35^ When an abstract is poorly reported or misinterpreted, clinicians make uninformed decisions and may disseminate biased results to colleagues, patients, and the media in general.^34^

Four of the 13 included studies investigated spin in SRs,^17,22,25,28^ and all of them found it. SRs are especially relevant because they aim to synthesize all the available evidence on a topic and occupy a prominent (top) place in the evidence hierarchy, being very influential in clinical practice. Therefore, spin in this study design is even more concerning. The four studies that assessed spin in SRs used the classification proposed by Yavchitz et al.^36^ in 2016. This tool identifies 13 types of spin in SRs and classifies them into three main categories: misleading reporting, defined as incomplete or inadequate reporting of methods, results, or study analysis; misleading interpretation, defined as an interpretation of study results to mislead the reader; and inappropriate extrapolation, where an inappropriate generalization of study results occurs. It also proposes a ranking to classify spin according to severity, where the most severe is the conclusion formulating recommendations for clinical practice not supported by the findings. The use of the tool standardizes the classification of spin, thus allowing editors and readers to identify it and reduce misinterpretation of the results of SRs.^36^

Although in 2010 Boutron et al.^1^ proposed a classification scheme for RCTs that has served as a basis for subsequent studies, no formal tool has been developed yet. Consequently, the studies included in the present scoping review that investigated spin in RCTs used diverse classification schemes developed by each study individually, thus hindering proper identification, classification, and comparison of the types of spin reported in these studies. Therefore, our findings suggest the need to develop a tool for classification of spin in RCTs.

The scientific community tends to give more value to statistically significant results, favoring their publication over statistically nonsignificant ones, which leads to selective outcome reporting.^37^ However, when it comes to clinical decision-making about treatments, any result is important, whether statistically significant or not. Publishing negative (or nonsignificant) results prevents both ineffective treatments from being applied and new studies on the same topic from being conducted, reducing wasteful research.^37^ Selective outcome reporting, i.e., post hoc changes in outcome reporting, can distort the interpretation of treatment effects, affect the validity of clinical trials, inflate effect size estimates in future meta-analyses, and, therefore, misguide treatment recommendations and policies. Thus, registering protocols prospectively is paramount to curb selective outcome reporting and to bring transparency and ethics to the research ecosystem.^38,39^

One approach to mitigate the occurrence of spin in studies is to strictly adhere to reporting guidelines such as PRISMA^40^ and CONSORT,^41^ which provide frameworks for reporting study results. However, it is important to note that following a checklist does not guarantee the absence of spin in authors’ reporting. Although the first version of PRISMA was published in 2009^40^ and of CONSORT in 1996,^41^ our review showed that studies with spin are currently being published in the dental field. The same is true for tools used to assess the risk of bias in RCTs, such as the Cochrane RoB 2 tool,^42^ and those used to assess the risk of bias in SRs, such as AMSTAR 2^43^ and ROBIS.^44^ However, assessing the risk of bias with these tools does not prevent spin from occurring, since the purpose of the tools is to evaluate the extent to which the methods employed in the study pose a threat to its validity rather than assessing the report itself. In other words, authors may have conducted a well-designed RCT that was deemed at low risk of bias, but they may still have added spin when reporting the results or conclusions, and this practice is not avoided by using the aforementioned tools.

Our study has some limitations. The topic of our study is very recent, and we believe that new studies are underway at the moment. Therefore, new results could change the conclusions of our review. In addition, since we are dealing with a topic that has been debated for a relatively short period of time, the indexing system is suboptimal. For example, in MEDLINE, there is still no MeSH term to define spin and MeSH terms for dental specialties are not exhaustive, with only eight specialties described. Therefore, a comprehensive and highly sensitive search strategy had to be developed because many studies were poorly indexed in the databases, which made it difficult to identify them through electronic searches. Conversely, a highly sensitive search allowed us to be confident that we have not missed potentially eligible studies.

## Conclusions

The practice of spin is widespread in dental scientific literature. It is present in several dental specialties, journals, and countries. Although the study of spin seems to be progressing, its impact remains poorly investigated. Further studies on the topic may increase awareness of spin among dental students, reviewers, editors, researchers, and clinicians in general. It is important to investigate the impact of spin on different outcomes, such as the understanding and persuasiveness of the studies’ results / conclusions and the decision-making process by health professionals.
